# Baseline levels and longitudinal rates of change in plasma Aβ42/40 among self-identified Black/African American and White individuals

**DOI:** 10.21203/rs.3.rs-3783571/v1

**Published:** 2024-01-08

**Authors:** Chengjie Xiong, Suzanne Schindler, Jingqin Luo, John Morris, Randall Bateman, David Holtzman, Carlos Cruchaga, Ganesh Babulal, Rachel Henson, Tammie Benzinger, Quoc Bui, Folasade Agboola, Elizabeth Grant, Gremminger Emily, Krista Moulder, David Geldmacher, Olivio Clay, Erik Roberson, Charles Murchison, David Wolk, Leslie Shaw

**Affiliations:** Washington University in St. Louis; Washington University School of Medicine; Washington University in St. Louis; Knight Alzheimer Disease Research Center; Washington University in St. Louis; Washington University in St. Louis; Washington University School of Medicine; Washington University School of Medicine; Washington University School of Medicine; Washington University in St. Louis; Washington University School of Medicine; Washington University School of Medicine; Washington University School of Medicine; Washington University School of Medicine; Washington University School of Medicine; University of Alabama Birmingham; University of Alabama Birmingham; University of Alabama at Birmingham; University of Alabama Birmingham; Department of Neurology, University of Pennsylvania; Perelman School of Medicine, University of Pennsylvania

## Abstract

**Objective:**

The use of blood-based biomarkers of Alzheimer disease (AD) may facilitate access to biomarker testing of groups that have been historically under-represented in research. We evaluated whether plasma Aβ42/40 has similar or different baseline levels and longitudinal rates of change in participants racialized as Black or White.

**Methods:**

The Study of Race to Understand Alzheimer Biomarkers (SORTOUT-AB) is a multi-center longitudinal study to evaluate for potential differences in AD biomarkers between individuals racialized as Black or White. Plasma samples collected at three AD Research Centers (Washington University, University of Pennsylvania, and University of Alabama-Birmingham) underwent analysis with C_2_N Diagnostics’ PrecivityAD^™^ blood test for Aβ42 and Aβ40. General linear mixed effects models were used to estimate the baseline levels and rates of longitudinal change for plasma Aβ measures in both racial groups. Analyses also examined whether dementia status, age, sex, education, *APOE ε*4 carrier status, medical comorbidities, or fasting status modified potential racial differences.

**Results:**

Of the 324 Black and 1,547 White participants, there were 158 Black and 759 White participants with plasma Aβ measures from at least two longitudinal samples over a mean interval of 6.62 years. At baseline, the group of Black participants had lower levels of plasma Aβ40 but similar levels of plasma Aβ42 as compared to the group of White participants. As a result, baseline plasma Aβ42/40 levels were higher in the Black group than the White group, consistent with the Black group having lower levels of amyloid pathology. Racial differences in plasma Aβ42/40 were not modified by age, sex, education, *APOE ε*4 carrier status, medical conditions (hypertension and diabetes), or fasting status. Despite differences in baseline levels, the Black and White groups had a similar longitudinal rate of change in plasma Aβ42/40.

**Interpretation:**

Black individuals participating in AD research studies had a higher mean level of plasma Aβ42/40, consistent with a lower level of amyloid pathology, which, if confirmed, may imply a lower proportion of Black individuals being eligible for AD clinical trials in which the presence of amyloid is a prerequisite. However, there was no significant racial difference in the rate of change in plasma Aβ42/40, suggesting that amyloid pathology accumulates similarly across racialized groups.

## 1 INTRODUCTION

Biomarkers of Alzheimer disease (AD), including fluid and imaging biomarkers of amyloid and tau pathology, have enabled a better understanding of AD pathophysiology, facilitated clinical trials that have led to the development of amyloid-lowering treatments, and increased the accuracy of clinical dementia diagnosis^1^. While cerebrospinal fluid (CSF)- and positron emission tomography (PET)-based biomarkers accurately detect AD brain pathology, the scale of testing with these modalities is limited by their requirements for specialized personnel and equipment, perceived risks, and high costs^1–3^. In contrast, blood is routinely collected in research and clinical care and considered highly accessible, acceptable, and scalable, making blood-based biomarkers ideal tools for research, clinical trials, and clinical practice^4, 5^. Therefore, blood-based biomarkers of AD may facilitate performing biomarker testing in minoritized groups that have historically been under-represented and systematically excluded in AD research and clinical trials^6–9^. Further, Black individuals with cognitive impairment may be less likely to be seen in memory clinics that perform biomarker testing with CSF and PET and serve as an entry point for research studies and clinical trials^10^. The use of blood-based biomarkers may enable testing of individuals in racialized groups who would not be willing to undergo screening with CSF or PET testing, and may enable testing in a community-based setting rather than a major medical center.

Multiple epidemiological studies have reported a higher prevalence of dementia in self-identified Black or African American and Hispanic individuals as compared to non-Hispanic White individuals (nHW)^7, 11–13^. Despite the higher reported prevalence of dementia, several research studies have reported a lower rate of AD biomarker abnormalities in Black and Hispanic individuals^14–20^, although other studies have found the opposite result or no differences between these groups^21^. The seeming disconnect between the reported prevalence of dementia and the rate of AD biomarker abnormalities has raised concern that the major etiologies of dementia may vary across racial and ethnic groups and/or that biomarkers may not reflect AD pathology consistently across groups, in addition to the possibility that the diagnostic evaluation for dementia may vary across studies. Further, concentrations of some plasma biomarkers can be affected by medical conditions (e.g., chronic kidney disease and obesity) that are more prevalent in some racial and ethnic groups, suggesting that plasma biomarkers may not reflect AD pathology consistently across groups^22–25^. However, some evidence indicates that plasma biomarker ratios may normalize for individual-level differences and provide more consistent performance across groups ^22,24–26^. Specifically, we have previously reported that plasma Aβ42/40 as measured by a high precision mass spectrometry-based assay has more consistent performance in classifying amyloid status across racial groups as compared to concentrations of phosphorylated tau ^22^. This finding suggests that plasma Aβ42/40 as measured by high precision assays may enable classification of amyloid status in more diverse groups.

Almost all studies of racial differences in AD biomarkers have been based on CSF and imaging biomarkers, and the few studies that have reported data on plasma biomarkers only reported cross-sectional data ^22, 27–30^. Therefore, it is unknown whether the longitudinal rates of change in plasma biomarkers vary by race or ethnicity. The rate of change is particularly important in clinical trials, as it represents the placebo trajectory of AD pathology that is intended to be modified by treatments. This study utilized one of the largest biracial cohorts with plasma biomarkers assembled thus far to evaluate for potential differences in baseline levels and rates of change in plasma Aβ measures (Aβ42, Aβ40, and Aβ42/40) in self-identified Black and White participants. Participants from three AD Research Centers (Washington University, University of Pennsylvania, and University of Alabama at Birmingham) were included. Samples were analyzed with the C_2_N Diagnostics mass spectrometry-based assay that is currently being used in clinical trials and clinical practice ^31–32^. General linear mixed effects models were used to estimate the baseline levels and rates of change for plasma Aβ measures in both racialized groups. Analyses also examined whether dementia status, age, sex, education, *APOE* ε4 carrier status, fasting status, and comorbidities (hypertension and diabetes) modified potential racial differences.

## 2 METHODS

### 2.1 Participants

The study cohort included individuals with plasma Aβ measures and clinical/cognitive data who participated in the Study of Race to Understand Alzheimer Biomarkers (SORTOUT-AB; NIH/NIA R01 AG067505), which aims to understand potential racial differences in harmonized biomarker data collected by multiple research studies of memory and aging in middle-aged and older individuals. Participants in the current study represented three of the SORTOUT-AB sites: the Washington University (WU) Knight Alzheimer Disease Research Center (ADRC), the University of Pennsylvania (UPenn) ADRC, and the University of Alabama at Birmingham (UAB) ADRC. Details of recruitment for these studies have been described previously^16,33^. Participants with conditions that could prevent participation or affect long-term participation (e.g., metastatic cancer) were excluded. Participants underwent clinical and/or cognitive assessments within 2 years of their baseline plasma assessments. A subset of the participants also had CSF or imaging assessments within 2 years of their baseline plasma sample collection. All participants provided written informed consent at recruitment from their parent studies. The Washington University Human Research Protection Office approved the current study with additional approvals from the Institutional Review Boards of the other sites.

### 2.2 Clinical and cognitive assessments

Clinical and cognitive assessments from WU, UPenn, and UAB followed protocols consistent with the National Alzheimer’s Coordinating Center Uniform Data Set (UDS)^34–35^. Demographic information, body mass index (BMI), and medical history were collected. Race and sex were self-identified by participants. The presence or absence of dementia, and when present, its severity, was determined by the score on the Clinical Dementia Rating^®™^ (CDR^®™^)^36^, which was performed at baseline and then annually. Standard criteria were used to diagnose the likely etiology of dementia^37^. The cognitive battery of the UDS included tasks of episodic memory, working memory, semantic knowledge, executive function and attention, and visuospatial ability, and were harmonized across UDS versions^38^: Montreal Cognitive Assessment [MoCA], Animal Fluency (60 seconds), Vegetable Fluency, Wechsler Adult Intelligence Scale (WAIS-R) Digit Symbol, Digit Span, Craft Story Immediate Recall), Craft Story Delayed Recall), Multilingual Naming Test, Free and Cued Selective Reminding, and Trail Making Test A and B. All scales were oriented such that a higher score indicated better cognition and converted to Z-scores using the baseline mean and standard deviation (SD) from the entire cohort. A global cognitive composite score was calculated by averaging Z-scores across all tests. An episodic memory composite score was calculated by averaging the Z-scores from the Craft Story-immediate and Craft Story-delayed tasks.

### 2.3 Apolipoprotein E genotyping

Apolipoprotein E (*APOE*) genotyping was performed as previously described^16^. Participants were classified as *APOE ε*4 carriers (one or two ε4 alleles) and non-carriers.

### 2.4 Blood and CSF collection and analysis

At WU, blood was collected at the time of lumbar puncture (fasted) or clinical assessment (non-fasted)^39^. Only non-fasted samples were collected at UPenn, and only fasted samples were collected at UAB. At WU, CSF samples (20–30 mL) were collected at 8 AM after overnight fasting by gravity drip, briefly centrifuged at low speed, and aliquoted into polypropylene tubes prior to freezing at − 80°C. CSF samples from participants enrolled at UAB and UPenn ADRCs were collected in accordance with protocols for the Alzheimer’s Disease Neuroimaging Initiative (ADNI)^40^. All plasma samples were analyzed at C_2_N Diagnostics with the PrecivityAD^™^ assay. Briefly, Aβ40 and Aβ42 were simultaneously immunoprecipitated from plasma via a monoclonal anti-Aβ mid-domain antibody^41^. Proteins were digested into peptides using LysN endoprotease. Liquid chromatography-mass spectrometry was performed on a Thermo Scientific Orbitrap Lumos Tribrid mass spectrometer interfaced with a nano-Acquity chromatography system^41^.

An automated immunoassay (LUMIPULSE G1200, Fujirebio, Malverne, PA) was used to measure CSF concentrations of Aβ40, Aβ42, total tau (t-tau), and tau phosphorylated at position 181 (p-tau181)^42–43^. A bridging subset of the CSF samples (n = 114) from the UPenn ADRC was selected to represent a wide range of values for all analytes and were run at the same time and with the same reagents as the WU samples to evaluate and adjust for systematic differences between the UPenn and WU sites. A linear regression model fitted on the values of the bridging samples was used to harmonize the CSF biomarker values between UPenn and WU^44^.

### 2.5 Imaging processing and analysis

Details of the structural brain MRI and amyloid PET protocols are provided elsewhere^39,45–46^, which followed a protocol consistent with that used by the ADNI. A standardized uptake value ratio (SUVR) with correction for partial volume effects was calculated for the FreeSurfer regions of interest (ROIs) for PiB, Florbetapir or Florbetaben^45^. The cerebellum was used as the reference region. A summary measure of amyloid burden was calculated using the averaged SUVR values in the lateral orbitofrontal, medial orbitofrontal, precuneus, rostral middle frontal, superior frontal, superior temporal, and middle temporal regions. To harmonize SUVR values across different tracers (PiB, Florbetapir or Florbetaben), values from the summary measure were converted into Centiloid units^47^, following previously published methods^47–48^.

### 2.6 Statistical analyses

The baseline characteristics of participants were summarized with the mean and SD for continuous variables or count and percentage for categorical variables. General linear models were implemented to evaluate for cross-sectional racial differences in levels of plasma Aβ measures using baseline data from either participants with only baseline plasma data or participants with longitudinal plasma data. These models included the main effects of self-identified race and the covariates of age, cognitive BMI, and comorbidity status (hypertension, stroke, and diabetes). Additional analyses included interactions of each variable with racial group to examine whether each of the covariates modified the racial differences. In the subset of participants with CSF and imaging biomarkers, plasma Aβ measures were correlated with the established AD biomarkers by Spearman correlations, and the correlations between racialized groups were compared by a two-sided standard normal test after the Fisher’s Z-transformation^49^. Because of the large number of comparisons in the correlations, we adjusted statistical significance for a False Discovery Rate (FDR)^50^ of 5%.

General linear mixed models were implemented to evaluate for racial differences in the rate of change of plasma Aβ measures^51^, specifically the random intercept and random slope models that assume linear growth patterns over time^52^. All models included race and a race by time interaction (time 0 = baseline), and the covariates of age, cognitive status (CDR 0 or > 0), sex, years of education, *APOE ε*4 status, and type of sample (fasting or non-fasting), BMI, and comorbidities (hypertension, stroke, and diabetes) as fixed effects. The annual rates of change between racialized groups were compared by a two-sided approximate Student t test whose degree of freedom was estimated by the Satterthwaite method. All models were implemented in Rstudio (version 2023.9.1.494 running R version 4.2.1) via the R package *lmerTest* (version3.1–3). We further assessed whether a linear trend fitted the longitudinal data well, and found no clear nonlinear longitudinal patterns, likely because of small number of plasma samples for most individuals.

## 3 RESULTS

### 3.1 Cohort characteristics at baseline

The research-based study cohort included a total of 324 Black and 1,547 White participants with plasma Aβ measures from at least one sample. A sub-cohort of 158 Black and 759 White participants had plasma Aβ measures from at least two samples with a mean interval between the first and last plasma sample of 6.62 (SD = 4.42) years. Participant characteristics at baseline are shown (Table 1). Black and White participants had similar ages at baseline (70.2 ± 8.6 and 70.5 ± 9.5 years, respectively, p = 0.26), but Black participants had a shorter average interval between their first and last plasma sample (5.11 ± 3.52 years) compared to White participants (6.93 ± 4.17 years; p < 0.0001). Black participants (72.2%) were more likely to be cognitively normal than White participants (66.4%; p = 0.041) at baseline, but there was no difference in the proportion carrying an *APOE* ε4 allele (45.1% versus 42.6%, p = 0.35). Most participants completed at least 12 years of education, with Black participants (15.3 ± 2.9 years, p = 0.002) completing slightly fewer years of education on average compared to White participants (15.8 ± 2.8 years). Black participants were more likely than White participants to be female (72.2% versus 53.5%, p < 0.0001). Black participants were more likely than White participants to have a history of hypertension (65.4% versus 42.3%, p < 0.0001) or diabetes (17.3% versus 6.0%, p < 0.0001). Black participants also had a higher average BMI (30.3 ± 6.23) than White participants (27.5 ± 5.16; p < 0.0001). A higher proportion of baseline biomarker data from Black participants were non-fasted (63.9%) than from White participants (34.4%; p < 0.0001). A subset of 110 Black and 1040 White participants had CSF biomarker data at baseline. A subset of 129 Black and 798 White participants had amyloid PET data at baseline.

### 3.2 Cross-sectional racial differences in plasma Aβ biomarkers

Cross-sectional analyses included baseline plasma Aβ measures from either participants with only baseline plasma data or participants with longitudinal plasma data. Groups of Black and White participants were compared with adjustment for age, sex, *APOE* ε4 carrier status, years of education, fasting status, BMI, cognitive status, and history of diabetes and hypertension (Table 2). There were no differences in the covariate-adjusted mean levels of Aβ42 between Black and White groups (25.54 pg/mL vs. 25.70 pg/mL, p = 0.96), but the Black group had a significantly lower level of plasma Aβ40 (166.10 vs. 187.72 pg/mL; p = 0.0004). As a result, the ratio of Aβ42 to Aβ40 (Aβ42/40) was significantly higher in the Black group (0.1214 vs. 0.1168; p < 0.0001). Further analyses examined whether racial differences in the adjusted mean levels of biomarkers were modified by cognitive status, sex, *APOE* ε4 carrier status, years of education, BMI, or age (Table 3). Some of the racial differences between Black and White participants were numerically larger in certain sub-groups, such as the Aβ42/40 ratio in the cognitively normal compared to the cognitively impaired group, and Aβ40 in the younger compared to the older group. However, there were no significant interactions between racial group and any of the covariates, suggesting that regardless of cognitive status, sex, years of education, BMI, or *APOE* ε4 carrier status, Black participants had a higher mean level of Aβ42/40 compared to White participants.

### 3.3 Racial differences in the association of plasma Aβ biomarkers with CSF and imaging biomarkers and cognitive scores

**Figure 1:**
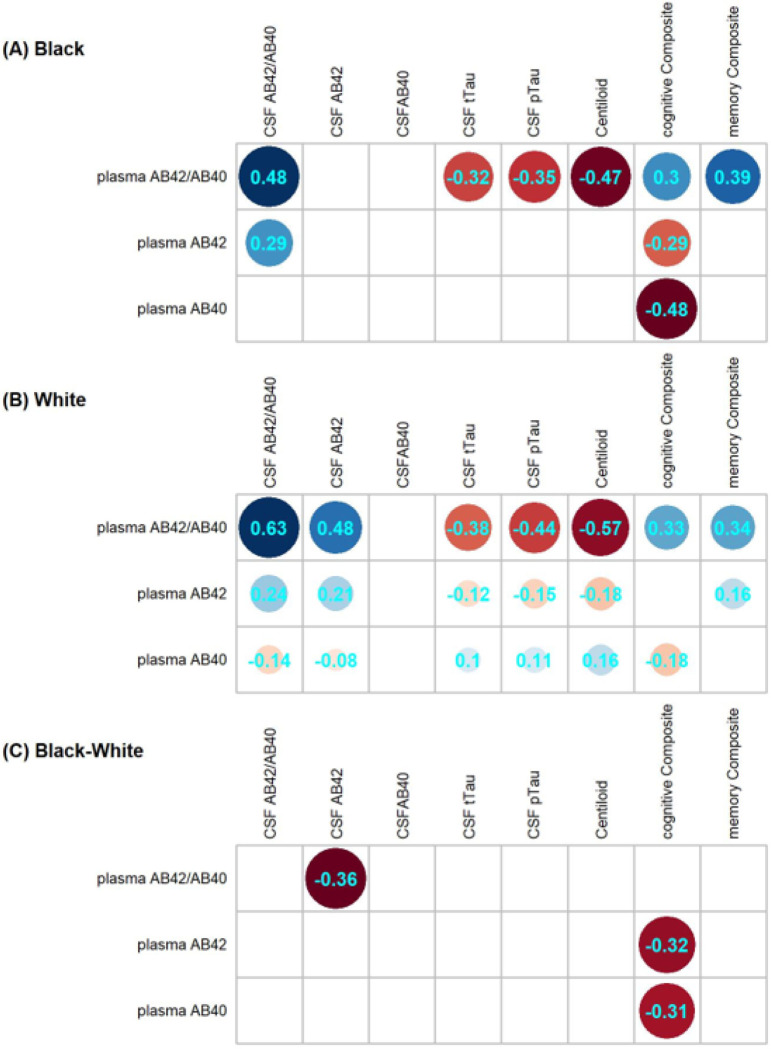
Spearman correlations of plasma biomarkers with CSF and imaging biomarkers and cognition and their differences between self-identified Black and White participants. Non-significant (raw P>0.05) correlation were made blank in Panel A and B. Non-significant differences in correlations (FDR P>0.05) were made blank in Panel C

Spearman correlations of plasma Aβ42, Aβ40, and Aβ42/40 with established CSF and imaging biomarkers and cognitive scores were examined within each racialized group and compared across Black and White groups (Fig. 1). Plasma Aβ42 and Aβ40 were only significantly correlated with a few measures in the Black group (likely due to lack of power), but were significantly correlated with almost all CSF and imaging biomarkers and cognitive scores in the larger White group. No significant racial differences were observed in correlations with plasma Aβ42 and Aβ40 except for the correlation between plasma Aβ42 and cognition (p = 0.0035, FDR p = 0.016) and between Aβ40 and cognition (p = 0.0027, FDR p = 0.015), in which Black participants had a stronger correlation. Plasma Aβ42/40 was correlated with CSF Aβ42/40 in both Black (r = 0.48) and White (r = 0.63) groups, and the difference was not significant (FDR p = 0.097). Plasma Aβ42/40 was negatively correlated with CSF total tau, CSF p-tau181, and amyloid PET Centiloid in both Black and White groups, and there were no racial differences in these correlations. Plasma Aβ42/40 was positively correlated with the global cognitive composite and episodic memory composite in both Black and White groups, and there were no racial differences in these correlations.

### 3.4 Racial differences in the longitudinal changes of plasma Aβ biomarkers

**Figure 2:**
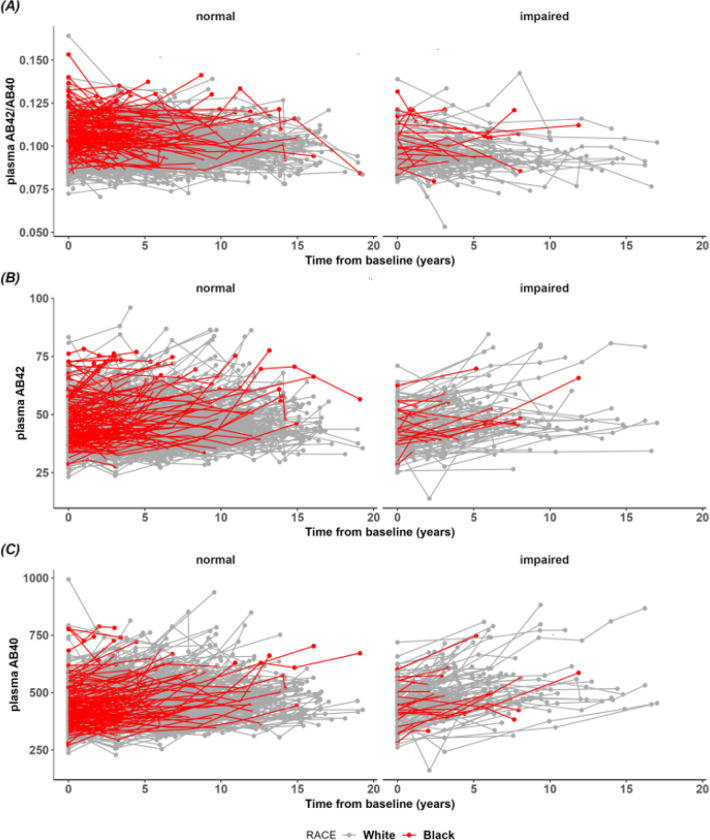
Spaghetti plots of plasma biomarkers against time since baseline between cognitively normal (CDR 0) and impaired (CDR>0) self-identified Black and White participants

Longitudinal trajectories of plasma Aβ42, Aβ40, and Aβ42/40 appeared relatively linear (Fig. 2). Black participants had a faster increase than White participants in plasma Aβ42 (Δ=0.31 pg/mL/year, SE = 0.12, p = 0.012). However, there was no difference between Black and White participants in the rate of change for plasma Aβ40 (Δ=1.89 pg/mL/year, SE = 1.13, p = 0.094). Further, there was no difference between Black and White participants in the rate of change for plasma Aβ42/40 (Δ=0.0001, SE = 0.0001, p = 0.35). For plasma Aβ42 and Aβ40, there was a significant interaction between racial group and baseline age such that younger but not older Black participants had a faster increase in Aβ42 (p = 0.0056) and Aβ40 (p = 0.018). For plasma Aβ42/40, there were no significant interactions between racial group and any covariates, suggesting that the rate of change in plasma Aβ42/40 is consistent across racial groups.

## 4. DISCUSSION

This study examined one of the largest biracial AD research cohorts assembled thus far to evaluate for potential differences in baseline levels and rates of longitudinal change in plasma Aβ measures (Aβ42, Aβ40, and Aβ42/40) in self-identified Black and White participants. We found that Black participants had a higher average baseline levels of plasma Aβ42/40 than White participants, which was due to lower average baseline levels of plasma Aβ40. Plasma Aβ42/40 was significantly correlated with almost all CSF and amyloid PET biomarkers as well as cognitive scores in White participants, and the correlations were largely consistent between Black and White participants. There were no significant racial differences in the rate of change in Aβ42/40 and Aβ40, but the Black group had a faster rate of increase in Aβ42 compared to the White group.

Our finding that Black research participants had higher average plasma Aβ42/40 levels, which is consistent with less amyloid pathology, aligns with three recent CSF and imaging biomarker studies^14–15,53^. One imaging study of 144 Black and 3,689 White cognitively normal individuals reported that the Black group had a lower rate of amyloid positivity and lower average amyloid burden ^15^. A second imaging study with 635 Black and 15,322 White cognitively impaired individual reported that Black participants were less likely to be amyloid PET positive^14^. In our own recent CSF biomarker study of 266 Black and 1,977 White participants, Black participants had less abnormality of multiple CSF biomarkers including CSF Aβ42/40, total tau, p-tau181, and neurofilament light^53^. In our current study, the lack of significant interactions between racial group and key covariates implies that racial differences in plasma Aβ measures are consistent across age, sex, *APOE* ε4 carrier status, BMI, and years of education. The consistency of racial differences despite adjustment for key covariates implies that racial group must be evaluated in statistical models analyzing plasma biomarker data. Notably, these biomarker differences, if confirmed by even larger studies on representative cohorts covering the entire spectrum of social determinants of health, may imply that a lower proportion of Black individuals will be eligible for research studies or clinical trials that use plasma biomarkers for amyloid positivity as inclusion criteria.

Despite racial differences in the average baseline levels of plasma Aβ42/40, this measure was correlated with most established CSF and amyloid PET biomarkers as well as cognitive composites in both racial groups. Further, the magnitude of correlations between plasma Aβ42/40 and other biomarker and cognitive measures were largely consistent between Black and White participants. One exception was the correlation between cognition and plasma Aβ42 and Aβ40, which was weaker in the White group. Studies with multiple high-accuracy plasma measures of amyloid pathology are needed to better understand potential differences in their relationships with cognitive outcomes.

A key finding of this study was that the rate of change in plasma Aβ42/40 did not vary significantly between groups of Black and White individuals, despite racial differences in baseline plasma Aβ42/40. This finding must be replicated and confirmed by even larger studies on representative cohorts covering the entire spectrum of social determinants of health. However, this finding suggests that while higher mean plasma Aβ42/40 levels may result in lower enrollment of Black participants in studies and trials that use biomarkers of amyloid pathology as inclusion criteria, once participants are enrolled and randomized, changes in plasma Aβ42/40 will likely be consistent across racial groups. Furthermore, the lack of interactions between racial group and key covariates in the rate of change implies that the rate of change in plasma Aβ42/40 is not differentially affected by these covariates. Since prevention and treatment trials follow participants to assess the efficacy of treatments, the consistency in rate of change may allow plasma Aβ42/40 to be used in biracial cohorts to establish the efficacy of treatments on biomarker change. Specifically, the placebo arm in future clinical trials may estimate the same rate of change in plasma Aβ42/40 across racial groups to which the active treatment arm may be compared to establish the biomarker efficacy of the treatment.

This study has multiple major strengths. Thus far, almost all previous studies of racial differences in AD biomarkers, including those with CSF, imaging, and blood-based biomarkers, were cross-sectional in nature^29,54–55^, and/or included relatively small numbers of Black participants who were typically enrolled at a single site. In contrast, this study included a relatively large number of Black participants with longitudinal plasma samples collected from three sites. Notably, this study used a plasma Aβ assay, PrecivityAD^™^, that was shown to accurately and consistently classify amyloid status in an overlapping biracial cohort ^22^. This test is currently being used in clinical trials as well as in clinical care ^32^, making our results of interest to researchers, clinical trialists and clinicians. Finally, significant correlations of plasma Aβ42/40 with CSF Aβ42/40 and amyloid PET demonstrates the potential value of plasma Aβ42/40 as a more acceptable and accessible biomarker of amyloid pathology. Limitations of our study include the limited data on structural and social determinants of health including socioeconomic status, especially life course experience and discrimination, that may correlate with biomarker measurements ^56^, and the fact that AD research cohorts are not representative of the general population^57–58^. Additionally, in our cohort we do not currently have data on plasma phosphorylated tau measures that have demonstrated very high accuracy in classifying amyloid status ^32^. Further, some negative findings must be interpreted with caution: the relatively large sample size of Black participants compared to other studies does not rule out that subtle racial differences may be present.

In summary, we found that Black research participants have higher average plasma Aβ42/40 at baseline, which may imply less amyloid pathology, compared to White participants. Interestingly, despite these racial differences at baseline, the rate of change of plasma Aβ42/40 was consistent in both Black and White groups. Further, plasma Aβ42/40 had consistent associations with CSF and imaging biomarkers as well as cognitive measures across racialized groups. These results suggest that plasma Aβ42/40 may be useful in providing a biomarker outcome for research and clinical trials that is consistent across racial groups.

## Figures and Tables

**Table 1:** Baseline demographics and clinical features

	White (N = 1547)	Black (N = 324)	P-value
**SITE**			< 0.0001
WashU	1412 (91.3%)	249 (76.9%)	
UPenn	115 (7.4%)	63 (19.4%)	
UAB	20 (1.3%)	12 (3.7%)	
**Baseline Age (yr)**			0.26
Mean (SD)	70.5 (9.53)	70.2 (8.60)	
Median [Min, Max]	71.0 [42.7, 98.0]	70.0 [43.5, 91.3]	
**Fasting status**			< 0.0001
fasting	1015 (65.6%)	117 (36.1%)	
non-fasting	532 (34.4%)	207 (63.9%)	
**CDR global**			0.065
0	1027 (66.4%)	234 (72.2%)	
0.5	377 (24.4%)	62 (19.1%)	
1	126 (8.1%)	20 (6.2%)	
2	16 (1.0%)	6 (1.9%)	
3	1 (0.1%)	1 (0.3%)	
Missing	0 (0%)	1 (0.3%)	
**Sex**			< 0.0001
M	720 (46.5%)	90 (27.8%)	
F	827 (53.5%)	234 (72.2%)	
**Education (yr)**			0.002
Mean (SD)	15.8 (2.81)	15.3 (2.92)	
Median [Min, Max]	16.0 [7.00, 29.0]	16.0 [6.00, 25.0]	
**APOE e4 positivity**			0.35
Negative	878 (56.8%)	172 (53.1%)	
Positive	659 (42.6%)	146 (45.1%)	
Missing	10 (0.6%)	6 (1.9%)	
**BMI (binary)**			< 0.0001
normal/underweight	426 (27.5%)	52 (16.0%)	
obese/overweight	805 (52.0%)	225 (69.4%)	
Missing	316 (20.4%)	47 (14.5%)	
**Hypertension**			< 0.0001
No	878 (56.8%)	108 (33.3%)	
Yes	655 (42.3%)	212 (65.4%)	
Missing	14 (0.9%)	4 (1.2%)	
**Stroke**			0.74
No	1401 (90.6%)	258 (79.6%)	
Yes	25 (1.6%)	6 (1.9%)	
Missing	121 (7.8%)	60 (18.5%)	
**Diabetes**			< 0.0001
No	1150 (74.3%)	223 (68.8%)	
Yes	93 (6.0%)	56 (17.3%)	
Missing	304 (19.7%)	45 (13.9%)	

**Table 2:** Cross-sectional estimates to plasma biomarker levels by race and their differences between self-identified Black and White participants, adjusting for the main effects of covariates

Marker	Group	Estimate	Standard Error	P
Aβ42/40	White	0.1168	0.0030	
	Black	0.1214	0.0030	
	Black-White	0.0046	0.0008	< 0.0001
Aβ42	White	25.6966	2.3829	
	Black	25.5411	2.4237	
	Black-White	−0.1555	0.6065	0.96
Aβ40	White	187.7185	22.5447	
	Black	166.0956	22.9315	
	Black-White	−21.6229	5.7385	0.00041

* adjusted for the main effects of age (continuous), sex (Male vs. Female), *APOE* ε4 carrier status (Positive vs. Negative), years of education (continuous), fasting status (fasted vs. non-fasted), cognitive status (CDR 0 vs. ≥0.5), BMI (obese/overweight vs. normal/underweight), and history of diabetes (Yes vs. No), stroke (Yes vs. No), and hypertension (Yes vs. No).

**Table 3:** Estimated cross-sectional differences and SE between Black and White participants in adjusted mean as a function of age (by median split at 70.62 y), sex, *APOE* ε4 carrier status, years of education, BMI, and cognitive status (CDR)

Plasma marker	Interacting covariate	Baseline adjusted mean difference (Black-White)	Standard Error	P value on baseline adjusted mean difference	P value on interaction*
Aβ42/40	CDR				0.0707
	= 0	0.0056	0.0009	4.26E-10	
	≥ 0.5	0.0017	0.0014	0.6315	
	*APOE* ε4				0.97
	negative	0.0043	0.0010	9.68E-05	
	positive	0.0049	0.0011	3.74E-05	
	Sex				0.999
	Male	0.0048	0.0013	0.001125	
	Female	0.0045	0.0009	3.80E-06	
	Age				0.999
	Younger	0.0046	0.0010	1.05E-05	
	Older	0.0045	0.0012	0.001	
	Education				0.999
	≤ 12 yr	0.0046	0.0015	0.0133	
	> 12 yr	0.0045	0.0009	4.20E-07	
	BMI				0.997
	≤ 30	0.0049	0.0017	0.0189	
	> 30	0.0045	0.0008	5.29E-07	
Aβ42	CDR				0.988
	= 0	−0.0451	0.6900	1.0000	
	≥ 0.5	−0.4841	1.1507	0.9761	
	*APOE* ε4				0.999
	negative	−0.0805	0.8054	1.000	
	positive	−0.2403	0.8522	0.993	
	Sex				0.997
	Male	0.0316	1.0354	1.0000	
	Female	−0.2455	0.7286	0.9878	
	Age				0.117
	Younger	−1.2192	0.7797	0.3888	
	Older	1.3430	0.9204	0.4525	
	Education				0.975
	≤ 12 yr	−0.6331	1.2182	0.9583	
	> 12 yr	−0.0319	0.6829	1.0000	
	BMI				0.210
	≤ 30	2.1860	1.3767	0.3597	
	> 30	−0.6702	0.6641	0.7281	
Aβ40	CDR				0.544
	= 0	−25.6400	6.5240	0.0004	
	≥ 0.5	−9.6669	10.8806	0.8005	
	*APOE* ε4				0.967
	negative	−19.2822	7.6194	0.0483	
	positive	−24.2676	8.0621	0.0123	
	Sex				0.996
	Male	−19.6700	9.7965	0.1673	
	Female	−22.5617	6.8933	0.0049	
	Age				0.103
	Younger	−31.9518	7.5148	0.0001	
	Older	−6.6313	8.8709	0.8817	
	Education				0.989
	≤ 12 yr	−25.0114	11.5203	0.12142	
	> 12 yr	−20.5383	6.4582	0.0071	
	BMI				0.261
	≤ 30	−0.8474	13.0271	1.0000	
	> 30	−26.1896	6.2839	0.0001	

* p value on the interaction between race group and the interacting covariate as indicated in the 2nd column.

# All results were adjusted for the main effects of covariates including age, sex, *APOE* ε4 carrier status, years of education, fasting status, cognitive status (CDR at baseline), BMI, and history of diabetes, stroke, and hypertension, but excluding the interacting covariate in the 2nd column.

**Table 4:** Estimated differences between self-identified Black and White participants in longitudinal rate of change (per year) as a function of baseline age (by median split at 70.62 y) and cognitive status (CDR 0 vs. >0), sex, *APOE* ε4 carrier status, BMI, and years of education adjusting for the main effects of all the other covariates

Plasma marker	Interacting covariate	Baseline adjusted mean difference (Black-White)	Standard Error	P value on baseline adjusted mean difference
Aβ42/40	CDR			0.466
	= 0	7e-05 (0.00016)	0.6499	
	≥ 0.5	0.00043 (0.00047)	0.3563	
	*APOE* ε4			0.0954
	negative	0.0003 (0.00019)	0.1109	
	positive	−0.00021 (0.00025)	0.3854	
	Sex			0.322
	Male	3e-04 (0.00024)	0.2157	
	Female	−1e-05 (2e-04)	0.9610	
	Age			0.401
	Younger	2e-04 (0.00018)	0.2552	
	Older	−8e-05 (0.00029)	0.7733	
	Education			0.213
	≤ 12 yr	0.00055 (0.00038)	0.1471	
	> 12 yr	4e-05 (0.00016)	0.8301	
	BMI			0.342
	≤ 30	−0.00029 (0.00043)	0.5016	
	> 30	0.00015 (0.00016)	0.3600	
Aβ42	CDR			0.975
	= 0	0.25649 (0.12978)	**0.0488**	
	≥ 0.5	0.2437 (0.380)	0.5215	
	*APOE* ε4			0.963
	negative	0.2499 (0.1547)	0.1070	
	positive	0.2618 (0.2015)	0.1944	
	Sex			0.199
	Male	0.4550 (0.1945)	**0.0200**	
	Female	0.1320 (0.1593)	0.4075	
	Age			**0.0056**
	Younger	0.4817 (0.1408)	**0.0007**	
	Older	−0.2707 (0.2333)	0.2463	
	Education			0.652
	≤ 12 yr	0.3834 (0.3080)	0.2139	
	> 12 yr	0.2318 (0.1339)	0.0843	
	BMI			0.391
	≤ 30	0.5236 (0.3526)	0.1380	
	> 30	0.2004 (0.1330)	0.1325	
Aβ40	CDR			0.682
	= 0	1.5650 (1.1910)	0.1896	
	≥ 0.5	0.0636 (3.4732)	0.9854	
	*APOE* ε4			0.266
	negative	0.4466 (1.4214)	0.7536	
	positive	3.0208 (1.8434)	0.1018	
	Sex			0.601
	Male	2.2056 (1.7892)	0.2186	
	Female	0.9987 (1.4582)	0.4937	
	Age			0.018
	Younger	3.3039 (1.2942)	0.0111	
	Older	−2.5717 (2.1290)	0.2275	
	Education			0.694
	≤ 12 yr	0.3853 (2.8301)	0.8918	
	> 12 yr	1.5981 (1.2307)	0.1949	
	BMI			0.152
	≤ 30	5.6108 (3.2231)	0.0823	
	> 30	0.6663 (1.2188)	0.5849	

* p value on the interaction between race group and the interacting covariate as indicated in the 2nd column.

# All results were adjusted the main effects of covariates including age, sex, *APOE* ε4 carrier status, years of education, fasting status, cognitive status (CDR at baseline), BMI, and history of diabetes, stroke, and hypertension but excluding the interacting covariate in the 2nd column.

## Data Availability

Anonymized data that support the findings of this study are available from the corresponding author and the first author, upon request from any qualified investigator.
